# Comparison of Different Treatment Outcomes for Refractory Overactive Bladder: A Systematic Review and Meta-Analysis

**DOI:** 10.3390/toxins17100479

**Published:** 2025-09-26

**Authors:** Maria Patricia Roman, Răzvan Ciortea, Stergios K. Doumouchtsis, Andrei Mihai Măluțan, Carmen Elena Bucuri, Cristina Mihaela Ormindean, Viorela Elena Suciu, Ionel Daniel Nati, Andreea Căilean, Dan Mihu

**Affiliations:** 1 2nd Obstetrics and Gynaecology Clinical Section, Cluj County Emergency Clinical Hospital, 55-57 21st of December 1989 Bld, 400124 Cluj-Napoca, Romania; 2 Mother and Child Department, “Iuliu Hațieganu” University of Medicine and Pharmacy, 8 Victor Babes Str, 400347 Cluj-Napoca, Romania; 3 Department of Obstetrics and Gynaecology, Epsom and St. Helier University Hospitals NHS Trust, Dorking Rd, Epsom KT187EG, UK; 4 Institute of Medical and Biomedical Education, St. George’s University of London, Cranmer Terrace, London SW17 0RE, UK; 5 Laboratory of Experimental Surgery and Surgical Research “N.S. Christeas”, Medical School, National and Kapodistrian University of Athens, 75 Mikras Asias Str, Goudi, 11527 Athens, Greece; 6 School of Medicine, American University of the Caribbean, Pembroke Pines, FL 33027, USA; 7 School of Medicine, Ross University, 10315 USA Today Way, Miramar, FL 33025, USA; 8 Military Emergency Hospital “Dr. Constantin Papilian”, 22 General Traian Moșoiu Str, 400132 Cluj-Napoca, Romania

**Keywords:** refractory overactive bladder, botulinum toxin, sacral neuromodulation, patient-reported outcomes, meta-analysis

## Abstract

Background: Refractory overactive bladder (OAB) poses a significant clinical burden, often severely impacting quality of life (QoL). While intradetrusor onabotulinumtoxinA (BoNT-A) and sacral neuromodulation (SNM) are established therapeutic options, a direct comparison of their efficacy and safety profiles is essential to guide clinical decision-making. This study compares BoNT-A against placebo and SNM for the management of refractory OAB in women. Methods: Following PRISMA guidelines, PubMed, Scopus, CENTRAL, and Google Scholar were searched until February 2025 for randomized controlled trials (RCTs) and cohort studies on treatment alternatives for refractory OAB. Treatment outcomes at 3- (BoNT-A vs. placebo) and 6-month (BoNT-A vs. SNM) follow-up were analyzed. Odds ratios (ORs) and mean differences (MDs) were calculated for dichotomous and continuous variables, respectively, with heterogeneity assessed via I^2^ test. Study quality was evaluated using CASP tools. Results: Pooled data from 12 studies (2645 patients) indicated that BoNT-A significantly reduced urgency urinary incontinence (UUI) episodes compared to placebo (*p* = 0.02) and SNM (*p* = 0.0008). Additionally, a ≥75% reduction in UUI episodes was more likely with BoNT-A compared to both placebo (*p* < 0.00001) and SNM (*p* < 0.00001). Complete resolution of UUI was more likely with BoNT-A compared to placebo (*p* < 0.00001); however, when compared to SNM, the latter demonstrated a higher rate of complete UUI resolution (*p* < 0.00001). Patient-reported QoL did not show significant differences between BoNT-A and SNM (*p* = 0.2). Urinary tract infection (UTI) risk was higher with BoNT-A than both comparators. Conclusions: While BoNT-A offers robust symptom control, its safety profile necessitates careful patient selection. SNM remains a viable alternative for those prioritizing fewer adverse events. The study highlights the need for standardized outcome reporting, long-term cost-effectiveness analyses, and personalized treatment approaches.

## 1. Introduction

Overactive bladder (OAB) is a common condition characterized by urgency, with or without urgency urinary incontinence (UUI), and may be associated with frequency and nocturia [1,2]. It occurs in the absence of any other identifiable pathologies such as urinary tract infections (UTI) [1,3]. The pathogenesis of OAB is complex, involving neurogenic, myogenic, and urothelial mechanisms [4,5], and is thought to result from impaired coordination and signaling between the nervous system and the bladder.

Estimates suggest that 12–14% of women are affected [6], with prevalence increasing with age [7,8]. The condition significantly impairs quality of life (QoL), affecting women’s physical well-being, emotional health, and their social, professional, and familial interactions.

Management of OAB is typically stratified by invasiveness, safety profile, and cost-effectiveness. First-line treatments include behavioral therapies, focused on lifestyle changes, bladder training, and pelvic floor muscle exercises [9]. Pharmacologic therapy such as anticholinergics and beta-3 adrenergic receptor agonists is offered when behavioral measures are insufficient. However, medication drop rates are often high due to side effects [10,11]. Approximately 40% of women experience treatment failure with conservative and pharmacologic options [6] and are therefore diagnosed with refractory OAB. In such cases, women may be offered minimally invasive options such as sacral neuromodulation (SNM) or intradetrusor onabotulinumtoxinA (BoNT-A) injections.

Beyond clinical efficacy and safety, cost and resource availability are critical factors influencing the choice of treatment for refractory OAB. SNM and intradetrusor BoNT-A injections differ significantly in costs, long-term cost-effectiveness, and accessibility, particularly in resource-limited settings [12]. While BoNT-A is less expensive and more widely available, SNM may offer durable benefits that justify its higher initial cost if therapeutic effects persist beyond 5 years [12]. However, comparative cost-effectiveness data reported in the literature remain sparse, underscoring the need for integrated financial evaluations in future studies.

Several systematic reviews have previously compared BoNT-A and SNM for refractory OAB in women (He et al., 2020; Lo et al., 2020; Yang et al., 2020; Liu et al., 2022) [13,14,15,16]. While these reviews provide important insights, their evidence base did not extend to the most recent RCTs, and their findings indicated variation across efficacy outcomes. Moreover, their scope often emphasized clinical endpoints, with less integration of patient-reported measures. These limitations highlight the need for an updated meta-analysis that incorporates the latest RCTs and provides a comprehensive synthesis of both clinical and patient-reported outcomes.

At the same time, the broader evidence base for refractory OAB treatment remains highly heterogeneous [17,18]. This heterogeneity, which complicates data synthesis and clinical guidance, arises from varying outcome definitions, measurement tools, and follow-up periods [18]. Furthermore, poorly reported long-term efficacy and safety data exacerbate the problem, underscoring a critical need for more standardized research [19,20].

This systematic review and meta-analysis aims to address this gap by comparing the efficacy and safety of BoNT-A with both placebo and SNM in women treated for refractory OAB, with assessments conducted at 3- and 6-month follow-up intervals. Moreover, this study aims to evaluate the quality and consistency of outcome reporting across the included randomized controlled trials (RCTs) and comparative cohort studies while also examining any available cost-effectiveness data that has been documented.

## 2. Results

**Figure 1:**
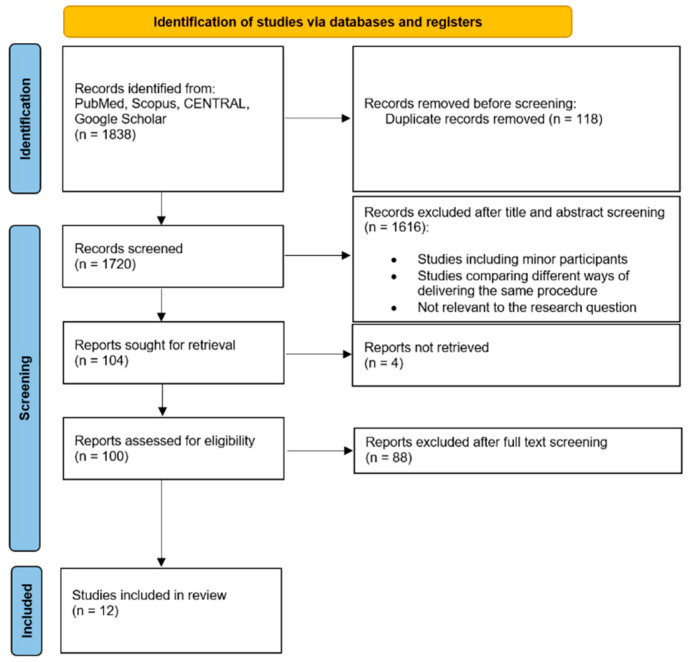
Study selection flowchart.

The PRISMA study selection flowchart [21] illustrates the systematic review process, detailing the initial identification of 1838 articles (Figure 1). After removing 118 duplicates, 1720 records were screened, with 1616 excluded based on titles and abstracts. Full-text assessment of the remaining 104 articles resulted in the exclusion of 92 studies due to ineligibility or non-retrieval, leaving 12 studies (9 RCTs and 3 cohort studies) for the meta-analysis, involving 2645 patients with refractory OAB.

The studies compared BoNT-A treatment with either placebo or SNM. Of the 12 included studies, 7 enrolled both male and female participants with female predominance (ranging from 75 to 93%), while the remaining 5 included exclusively female participants. Key characteristics of the included studies are summarized in Table 1.

The following sections present key findings of this meta-analysis, focusing on efficacy and safety outcomes. Consistency across outcomes was evaluated using the I^2^ test, and revealed generally low heterogeneity, with an I^2^ value below 50% in the majority (70.83%) of the analyses conducted.

### 2.1. BoNT-A vs. Placebo

#### 2.1.1. Efficacy Outcomes

**Figure 2:**
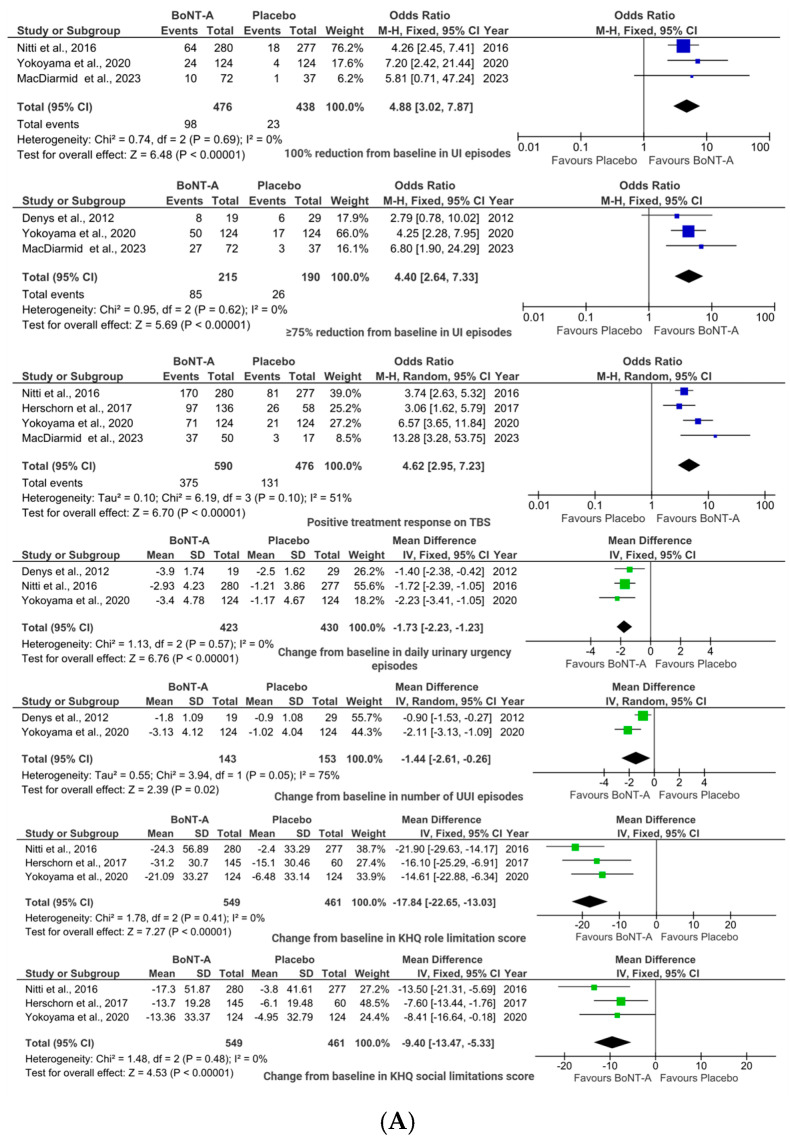

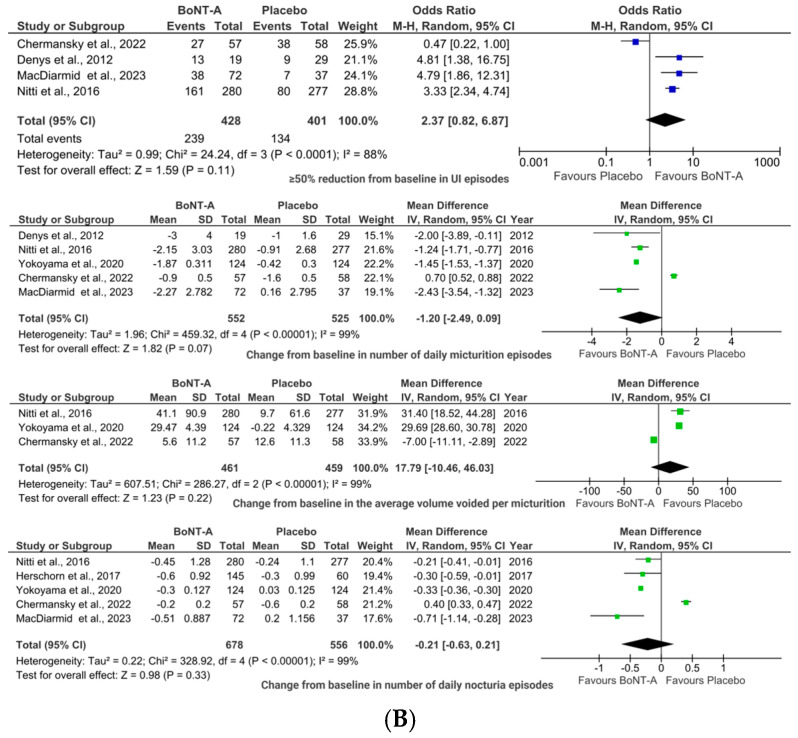
BoNT-A vs. placebo: forest plots illustrating pooled ORs or MDs with 95% confidence intervals for efficacy outcomes with (**A**) statistically significant differences and (**B**) statistically non-significant differences [24,25,29,30,31,32].

**Figure 3:**
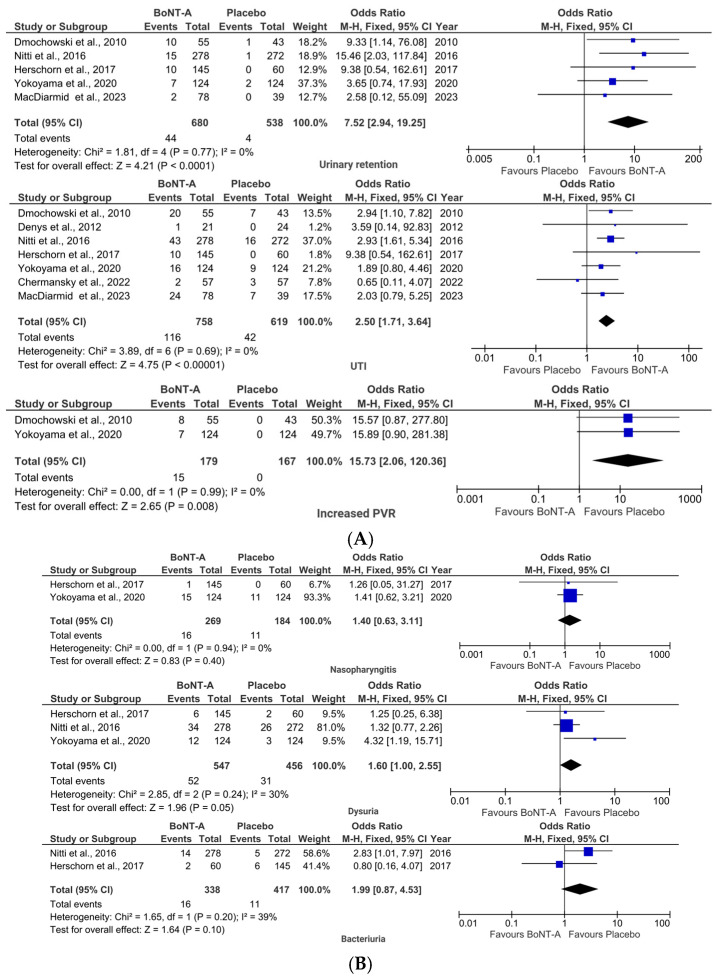
BoNT-A vs. placebo: forest plots illustrating pooled ORs for safety outcomes with (**A**) statistically significant dif-ferences and (**B**) statistically non-significant differences [24,25,26,29,30,31,32,33].

Pooled analysis revealed that a significant benefit of BoNT-A was seen in UI reduction, where the toxin achieved complete resolution of incontinence episodes in a significantly higher proportion of patients as compared to placebo (20.59% vs. 5.25%), OR 4.88, 95% CI (3.02, 7.87); *p* < 0.00001; (Figure 2A). More than one-third of patients (39.53% vs. 13.68%) experienced dramatic symptom improvement (≥75% reduction in urinary incontinence (UI) episodes, OR 4.40, 95% CI (2.64, 7.33); *p* < 0.00001; Figure 2A) with BoNT-A, while more modest reductions (≥50%) in UI episodes did not reach statistical significance compared to placebo (55.84% vs. 33.42%; OR 2.37, 95% CI (0.82, 6.87); *p* = 0.11; Figure 3A). Moreover, BoNT-A treatment showed no significant impact on daily micturition frequency (MD −1.20, 95% CI (−2.49, 0.09); *p* = 0.07; Figure 2B), voided volumes (MD 17.79, 95% CI (−10.46, 46.03); *p* = 0.22; Figure 2B), or nocturia episodes (MD −0.21, 95% CI (−0.63, 0.21); *p* = 0.33; Figure 2B).

Patient-reported outcomes significantly favored BoNT-A, with 63.56% vs. 27.52% of patients rating their treatment response as positive on the Treatment Benefit Scale (TBS) (OR 4.62, 95% CI (2.95, 7.23); *p* < 0.00001; Figure 2A). BoNT-A injections significantly reduced daily urgency episodes (MD −1.73, 95% CI (−2.23, −1.23); *p* < 0.00001; Figure 2A) and UUI events (MD −1.44, 95% CI (−2.61, −0.26); *p* = 0.02; Figure 2A). These improvements translated into significant QoL benefits for women receiving BoNT-A, particularly in King’s Health Questionnaire (KHQ) role limitations (MD −17.84, 95% CI (−22.66, −13.03); *p* < 0.00001; Figure 2A) and social limitations (MD −9.40, 95% CI (−13.47, −5.33); *p* < 0.00001; Figure 2A).

#### 2.1.2. Safety Outcomes

The therapeutic benefits came with several statistically significant adverse effects. Urinary retention occurred in significantly more (6.47%) BoNT-A patients vs. 0.74% controls (OR 7.52, 95% CI (2.94, 19.25); *p* < 0.0001; Figure 3A). Similarly, increased post-void residual volumes were more common in the BoNT-A group (OR 15.73, 95% CI (2.06, 120.36); *p* = 0.008; Figure 2B). UTI were more frequent in the BoNT-A group (15.3% vs. 6.79%; OR 2.50, 95% CI (1.71, 3.64); *p* < 0.00001; Figure 3A), though bacteriuria rates were comparable (4.73% vs. 2.64%; OR 1.99, 95% CI (0.87, 4.53); *p* = 0.10; Figure 3B).

However, only three/seven studies reported antibiotic use. Yokoyama et al. [29] and MacDiarmid et al. [31] reported antibiotics administration 1–3 days before, on the day of, and/or 1–3 days after BoNT-A injection, though without specifying agents or doses in all cases. Denys et al. [25] noted a single oral dose 90 min pre-injection. The remaining four studies by Chermansky et al., Dmochowski et al., Herschorn et al., and Nitti et al. [24,26,30,32] omitted antibiotic details entirely.

Other adverse events showed mixed patterns. Nasopharyngitis rates were similar between BoNT-A and placebo groups (5.95% vs. 5.98%; OR 1.40, 95% CI (0.63, 3.11); *p* = 0.4; Figure 3B). Although dysuria trended higher with BoNT-A (9.51% vs. 6.8%; OR 1.60, 95% CI (1.00, 2.55); *p* = 0.05; Figure 3B), statistical significance between groups was not reached.

#### 2.1.3. Sensitivity Analyses

Sensitivity analyses revealed that heterogeneity in “Positive treatment response on TBS” was largely driven by two studies (Yokoyama et al. (2020) [29]; MacDiarmid et al. (2023) [31]). Excluding either study reduced I^2^ from 51% to 43%, while the overall effect size remained consistent, supporting the robustness of the pooled estimate. Additional heterogeneity was observed for the outcomes “≥50% reduction from baseline in UI episodes” and change from baseline in “number of daily micturition episodes”, “the average volume voided per micturition”, and “in number of daily nocturia episodes”. In each case, heterogeneity was largely attributable to Chermansky et al. (2023) [24]. Exclusion of this study reduced I^2^ below 50% and altered the pooled effect to reach statistical significance (*p* < 0.05).

Following the comparison of BoNT-A with placebo, the efficacy and safety of BoNT-A were further assessed against SNM.

### 2.2. BoNT-A vs. SNM

The current meta-analysis revealed clinically important differences between intradetrusor BoNT-A injections and SNM for refractory overactive bladder in adult women.

#### 2.2.1. Efficacy Outcomes

**Figure 4:**
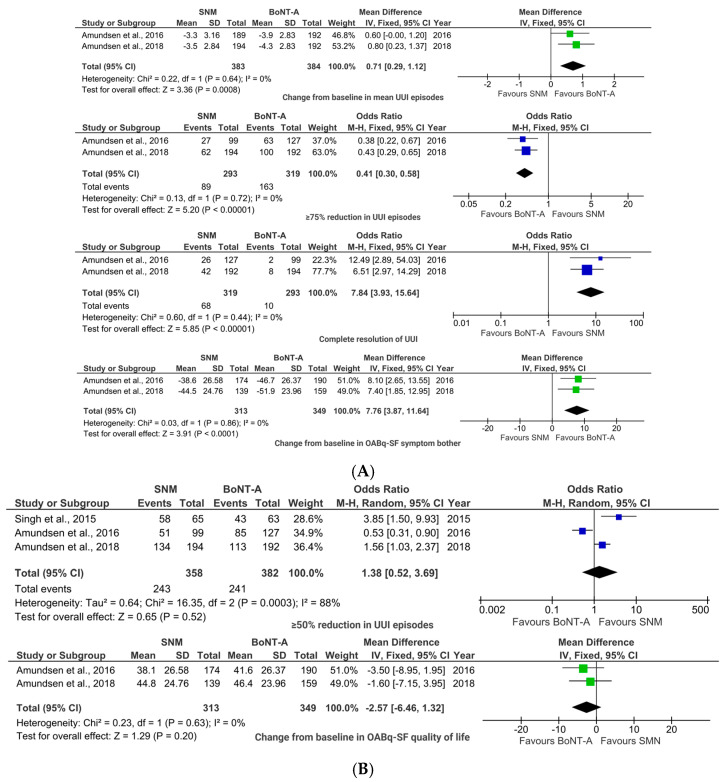
BoNT-A vs. SNM: forest plots illustrating pooled ORs or MDs for efficacy outcomes with (**A**) statistically significant differences and (**B**) statistically non-significant differences [22,23,28].

BoNT-A demonstrated superior efficacy for UUI compared to SNM. The pooled analysis showed significantly greater reductions in the mean number of UUI episodes (MD 0.71, 95% CI (0.29, 1.37); *p* = 0.0008; Figure 4A) for patients in the BoNT-A group. Additionally, significantly more patients in the BoNT-A group achieved ≥75% UUI reduction (OR 0.41, 95% CI (0.30, 0.58); *p* < 0.00001; Figure 4A). In contrast, the SNM group showed a significantly higher rate of complete UUI resolution (OR 7.84, 95% CI (3.93, 15.64); *p* < 0.00001; Figure 4A).

The ≥50% reduction threshold in UUI episodes did not show a statistically significant difference between study groups (OR 1.38, 95% CI (0.52, 3.69); *p* = 0.52; Figure 4B).

Patient-reported outcomes revealed that BoNT-A provided significantly greater improvement in OABq—short form (SF) symptom bother scores (MD 7.76, 95% CI (3.87, 11.64); *p* < 0.0001; Figure 4A), though OABq-SF QoL improvements were similar between groups (MD −2.57, 95% CI (−6.46, 1.32); *p* = 0.20; Figure 4B).

#### 2.2.2. Safety Outcome

**Figure 5:**
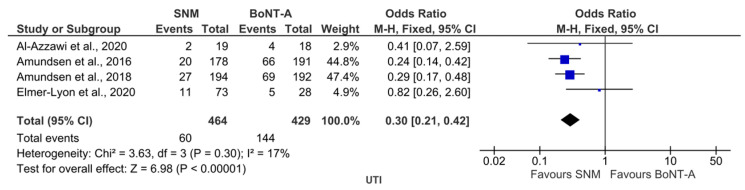
BoNT-A vs. SNM: forest plots illustrating the pooled ORs for the only safety outcome that showed a statistically significant difference [12,22,23,27].

The analysis revealed an important safety difference, with BoNT-A associated with significantly higher UTI rates (OR 0.30, 95% CI (0.22, 0.42); *p* < 0.00001; Figure 5).

Four out of five studies mentioned use of prophylactic antibiotics, but regimens differed. Amundsen et al. [22,23] reported use of oral ciprofloxacin (500 mg) immediately post-BoNT-A injection, as well as 3 days after the procedure and i.v. cefazolin (or clindamycin for penicillin allergy) pre-SNM. Elmer-Lyon et al. [27] stated that antibiotics were given post-procedure without further details. Singh et al. [28] explicitly reported avoidance of routine antibiotic prophylaxis, while Al-Azzawi et al. provided no data on antibiotics use.

#### 2.2.3. Sensitivity Analyses

Sensitivity analyses yielded that heterogeneity in “≥50% reduction in UUI episodes” outcome was mostly generated by the study conducted by Amunsden et al. (2016) [22]. However, excluding this study did not reduce I^2^ below the 50% threshold, and maintained the pooled effect stability.

### 2.3. Risk of Bias Assessment

#### 2.3.1. The Critical Appraisal Skills Programme (CASP) Assessment of RCTs [34]

Table 2 shows that 6 out of 13 CASP checklist items [34] were fully met by all included RCTs. Three CASP criteria had unclear (“ct”) ratings and referred to blinding of assessors, benefits vs. harms, and intervention value. The five CASP criteria that were not met pertain to participant follow-up, blinding, reported precision estimates, and intervention value. It is worth mentioning that in studies comparing BoNT-A with SNM, participant blinding was not feasible due to the distinct procedural differences between the treatments.

Most CASP checklist items (12/13) were fulfilled by two studies [31,32]. Four studies [24,26,29,30] met 11/13 items, one study [25] met 10/13 items, and the remaining two RCTs [22,23] met the fewest number of items (8/13).

#### 2.3.2. CASP Cohort Assessment [35]

Table 3 presents the quality assessment for the included cohort studies. Six CASP checklist items were fully met by all the included cohort studies. The criteria that were not met refer to six checklist items related to participants’ recruitment process, bias minimization, confounders, length of follow-up, and comparison of results with other available evidence.

None of the cohort studies met all the CASP criteria. The study conducted by Singh et al. [28] met most of the CASP criteria (10/12), while the other two cohort studies (al-Azzawi et al. [12] and Elmer-Lyon et al. [27]) each met 8/12 criteria. Table 3 provides full details of CASP items for cohort studies.

## 3. Discussion

### 3.1. Main Findings

This systematic review and meta-analysis comparing treatments for refractory OAB in women provides evidence on the efficacy and safety of intravesical BoNT-A vs. placebo and SNM.

#### 3.1.1. BoNT-A vs. Placebo

Placebo-controlled trials provide the highest level of evidence for evaluating the intrinsic efficacy and safety of an intervention [36]. Therefore, the placebo-controlled analysis was conducted to establish the standalone efficacy of BoNT-A in recently published studies, by isolating its effects from natural disease progression or placebo responses.

The key findings indicate that BoNT-A significantly outperforms placebo in reducing UI episodes, with a fourfold greater likelihood of achieving complete UI resolution as well as substantial reduction (≥75%) of UI episodes. Beyond documenting overall reductions in UI episodes, our study further categorized changes in UI symptoms at 50%, 75%, and 100% thresholds, offering a more detailed perspective that can assist in pre-intervention patient counselling, a feature not reported in other meta-analyses [37,38].

These objective measures are corroborated by PROMs, where over twice as many patients reported treatment satisfaction (TBS) with BoNT-A. In addition, the significant reductions in daily urgency episodes and UUI events further substantiate the toxin’s clinical value, translating to meaningful QoL improvements as evidenced by KHQ scores. Our results are consistent with prior systematic reviews that reported similar treatment outcomes [37,39].

While demonstrating robust efficacy, BoNT-A showed an adverse event profile requiring careful patient selection [40]. The sevenfold increased risk of urinary retention matches pooled data from Abrar et al.’s systematic review [19], with corresponding elevations in post-void residual volumes. The elevated UTI risk is comparable to the FUTURE trial report by Abdel-Fattah et al. in 2025 [6]. These findings suggest that patients with borderline voiding function or recurrent UTI may require alternative therapies or closer post-treatment monitoring.

Interestingly, the safety profile shows important nuances. First, the similar rates of bacteriuria (4.73% vs. 2.64%) may be attributed to the use of prophylactic antibiotics—a common practice before BoNT-A treatment [41]. However, the absence of standardized antibiotic reporting limits our ability to draw firm conclusions regarding bacteriuria and its clinical significance. Second, the absence of a difference between BoNT-A and placebo in terms of micturition frequency and voided volumes suggests that patients may intuitively adjust their voiding patterns (e.g., voiding more consciously, with abdominal straining) to ensure adequate bladder emptying after the procedure. Finally, non-urological adverse events, otherwise rarely reported, (e.g., nasopharyngitis) showed no significant difference, supporting good general tolerability.

The current pooled analyses contribute to the growing evidence on the effects of BoNT-A in treating refractory OAB [42,43]. In addition to the stratified analysis of UI symptom changes, this meta-analysis stands out by incorporating PROMs, including the TBS and KHQ, which are infrequently pooled in other meta-analyses, offering a more thorough evaluation of patient experiences and treatment satisfaction.

The placebo-controlled results reinforce the clinical significance of BoNT-A for women with refractory OAB; they demonstrate not only measurable efficacy but also improvements that are perceived as meaningful by patients in their daily lives. In addition, the alignment between objective outcomes and patient-reported measures strengthens the validity of these findings and underscores the real-world value of this intervention. Looking ahead, these results raise hope that BoNT-A can be more widely adopted and optimized in routine care, including in low-resource settings. This would allow expansion of treatment opportunities for women whose symptoms remain uncontrolled with standard therapies.

#### 3.1.2. BoNT-A vs. SNM

Although SNM is a widely used, guideline-recommended therapy for refractory OAB, its comparative effectiveness against BoNT-A remains debated. While both treatments modulate neural pathways, their mechanisms, invasiveness, and adverse event profiles differ. This comparison seeks to assess whether one procedure outperforms the other in key outcomes (e.g., symptom resolution, QoL) or if they are viable alternatives for specific patient subgroups.

For the comparison with the active comparator, BoNT-A demonstrated superior efficacy over SNM in reducing daily UUI episodes and achieving ≥75% reduction in UUI episodes. Our study indicated that BoNT-A also improved symptom bother (OABq-SF scores) more than SNM, though QoL outcomes were similar, possibly reflecting SNM’s lower side effect profile, which has been previously documented in the literature [33,44]. The lack of significant difference in ≥50% UUI reduction suggests both treatments are similarly effective for moderate responders, a finding that mirrors Niu et al.’s meta-analysis [33].

Surprisingly, while the toxin achieved a ≥75% reduction in UUI episodes, pooled analysis revealed that complete symptom resolution occurred more frequently with SNM. This unexpected finding contrasts with a prior meta-analysis [33]. The discrepancy could be due to variability in toxin dosing protocols, where higher doses may be more effective, or from expectation bias. Beyond that, the invasive nature of SNM (surgical implantation) might select for more motivated or adherent patients, which could indirectly improve outcomes.

In terms of safety, BoNT-A was associated with a significantly higher UTI risk, aligning with Lo et al.’s network meta-analysis on alternative treatment modalities for refractory OAB [15]. This higher UTI risk confirms findings from a recent critical review (2024), which mentioned UTI rates as high as 36% after BoNT-A injection vs. 15% with SNM [45]. While this risk may be acceptable for highly symptomatic patients with preserved voiding, SNM may be preferable for those at higher infection risk or unable to perform catheterization.

Our analysis also revealed that BoNT-A resulted in greater improvements in symptom bother, as measured by the OABq-SF. However, OABq-SF QoL scores did not differ significantly between groups. This suggests that while BoNT-A may be more effective in alleviating specific symptoms, SNM may offer comparable benefits in broader aspects of patient-reported QoL. A possible explanation is that SNM, despite more modest effects on UUI, avoids some of the side effects associated with BoNT-A, potentially mitigating the impact of persistent symptoms on daily function and well-being.

Considering these findings, it is interesting to highlight the trade-off between efficacy and tolerability. These results reinforce the idea that neither therapy is universally “better”; instead, the optimal choice depends on whether the individual patient prioritizes maximized symptom improvement or a safer, more tolerable long-term option. Looking forward, refinements in dosing strategies, treatment protocols, and more standardized reporting in future studies may help to more clearly delineate the relative safety and efficacy profiles of each procedure.

Our study’s results expand upon prior syntheses by providing integrated comparisons that not only offer stronger evidence to guide therapeutic decision-making but also address a critical gap in the literature, where choosing between BoNT-A and SNM remains challenging.

#### 3.1.3. Sensitivity Analyses

Sensitivity analyses confirmed the robustness of pooled estimates in outcomes such as “Positive treatment response on TBS” and “≥50% reduction in UUI episodes”. In contrast, other outcomes (e.g., “≥50% reduction from baseline in UI episodes” and change from baseline in “micturition episodes”, “voided volume”, and “nocturia episodes”) were sensitive to the inclusion of a single study [24], underscoring the importance of cautious interpretation of these findings. This finding may suggest that variations in study design, such as differences in administration protocols, may substantially alter pooled results, highlighting the need for standardized approaches to treatment delivery in future research.

### 3.2. Strengths and Limitations

This systematic review and meta-analysis provides an up-to-date synthesis of outcomes of minimally invasive treatments for refractory OAB in women. It includes both placebo-controlled and active comparator studies, enabling a comprehensive assessment of clinical efficacy and safety of tested treatments. The inclusion of both RCTs and comparative cohort studies enhances generalizability of findings. Methodological rigor was ensured through quality assessments conducted using the CASP tools [34,35], a framework widely recognized and endorsed by Cochrane and the World Health Organization [46,47,48]. The use of CASP tools to separately appraise the quality of RCTs and cohort studies adds transparency and robustness to the evaluation of included evidence.

In addition, the review offers detailed stratification of outcomes by intervention type and follow-up interval, improving the interpretability of results despite inter-study variability. Moreover, by prioritizing data from the guideline-recommended 100 IU BoNT-A dose (and 200 IU where relevant), this study enhances homogeneity and ensures alignment with clinical practice standards.

The study also identified underreported parameters such as PROMs, long-term follow-up, and cost-effectiveness data, thereby informing priorities for future trial design and outcome standardization in refractory OAB research.

Despite these strengths, several limitations should be acknowledged.

Variable follow-up intervals across studies may introduce confounding. While studies comparing BoNT-A with placebo consistently reported outcomes at 3 months, those comparing BoNT-A with SNM mostly reported 6-month follow-ups. Clinical experience suggests that while SNM effects stability by 6 months, BoNT-A’s benefits may start decreasing around this time [49]. However, our comparisons remain valid, as existing evidence confirms that the toxin’s clinical effects typically persist for 6–12 months, with longer duration observed at higher doses [32,50,51]. This temporal mismatch in peak effect periods, combined with the lack of standardized assessment windows, highlights the necessity for harmonized follow-up protocols that reflect both short- and long-term efficacy and safety profiles.

In addition to timing inconsistencies, the lack of standardized cost-effectiveness analyses in the included articles precluded economic comparisons, limiting our ability to assess the financial impact of BoNT-A vs. SNM. Moreover, differences in outcome measurement also limited comparability. While some studies [22,23,24] assessed PROMs (e.g., the Overactive Bladder Questionnaire (OABq), Incontinence Impact Questionnaire—short form (IIQ-7), Urinary Distress Inventory, short form (UDI-6)) or urodynamic parameters, inconsistent reporting, such as narrative results, p-values without effect sizes, or incomplete statistical data, precluded their inclusion in meta-analyses. This limitation reflects broader heterogeneity in outcome measurement across refractory OAB research.

Additionally, the variability in prophylactic antibiotic use and the lack of standardized antibiotic reporting prevents definitive conclusions about UTI risk associated with different procedures. This inconsistency may confound the observed UTI rates and highlights the need for future trials to standardize and transparently report antibiotic protocols.

Finally, given that some of the included studies were industry-funded [24,26,29,30,31], potential bias arising from industry sponsorship must be considered. However, the consistency of findings across studies minimizes this concern to some extent.

### 3.3. Implications for Practice

This study consolidates fragmented evidence into actionable insights for clinicians managing refractory OAB, balancing efficacy and safety outcomes of different treatment modalities. It confirms that intradetrusor BoNT-A injections are highly effective for reducing symptoms of refractory OAB in women, offering superior outcomes compared to both placebo and SNM at 3 and 6 months post-treatment, respectively. However, the increased risk of adverse events, particularly UTI, necessitates careful patient selection and monitoring. SNM remains a valuable alternative for those seeking an alternative option with a more favorable safety profile.

In addition to efficacy and safety outcomes, the cost implications of BoNT-A and SNM warrant careful consideration. However, only one of the included studies reported data on cost efficiency [8]. SNM is associated with higher upfront costs due to device expenses, surgical implantation, and follow-up requirements, whereas BoNT-A involves recurrent expenses for repeat injections every 6–12 months. Regional disparities further complicate accessibility; for example, SNM is unavailable in some countries (e.g., Iraq) and limited by a lack of trained specialists [8]. Despite its higher initial cost, SNM may become cost-effective if its therapeutic effect exceeds 5 years, given its broader indications and durability. Conversely, BoNT-A remains a pragmatic alternative in settings where SNM is prohibitively expensive or inaccessible, though its long-term cost burden and higher UTI risk must be weighed.

This study underscores the importance of personalized treatment choice and highlights gaps in the literature, such as the need for standardized outcomes and outcome measures, long-term efficacy data, and comprehensive cost evaluations. Healthcare systems should prioritize cost-effectiveness analyses to guide policy decisions, particularly in regions with limited resources. Patient-specific factors such as financial constraints, local expertise, and device availability should be central to shared decision-making.

By integrating these findings into clinical practice, providers can better tailor interventions to meet the diverse needs of women with refractory OAB, ultimately improving their QoL and treatment satisfaction.

## 4. Conclusions

In conclusion, this meta-analysis provides evidence that BoNT-A injections offer superior efficacy over SNM in achieving substantial (≥75%) UUI reduction and improvement in patient-reported symptom bother. However, these benefits are offset by a higher incidence of UTI. Therefore, treatment choices should be personalized. This involves balancing the intervention’s efficacy and adverse events with the patient’s own risks, preferences, and contextual factors. Future research must address the gap in cost-effectiveness data, with standardized comparisons of long-term expenses (e.g., repeat BoNT-A injections vs. SNM device longevity) across diverse healthcare systems. Until such evidence is available, treatment selection must rely on a holistic consideration of clinical outcomes and the individual patient context.

## 5. Materials and Methods

### 5.1. Registration and Data Sources

This study was registered in the international prospective register of systematic reviews (PROSPERO) [52] under the registration number CRD420250646524 and conducted in accordance with the Preferred Reporting Items for Systematic Reviews and Meta-Analyses (PRISMA) guidelines [53].

Multiple databases including PubMed, Scopus, Cochrane Central Register of Controlled Trials (CENTRAL), and Google Scholar were searched from their inception to 25th February 2025 to identify relevant articles. The following search terms were used: “refractory overactive bladder” AND (“female” OR “women” OR “woman”) AND (“randomised controlled trial” OR “randomized controlled trial” OR “randomised trial” OR “randomized trial” OR “cohort”). Duplicate records were identified and removed. To identify additional relevant articles that may have been missed in the initial search, we conducted handsearching of reference lists from primary studies, a technique known as snowballing. The searches were restricted to articles published in English, but no limitations were applied based on cultural or ethnic background.

### 5.2. Inclusion and Exclusion Criteria

Studies were included if the following criteria were met: they were RCTs or comparative cohort studies investigating treatment options for idiopathic refractory OAB, included ≥75% adult female participants, and used quantitative methodology.

Studies were excluded for the following reasons: they used mixed or non-quantitative methodology, evaluated the same treatment administered differently, were systematic reviews, case reports, educational articles, or included minor participants.

While our primary interest was in treatment outcomes in females, strict exclusion of all studies with male participants would have significantly reduced the number of eligible studies. This could have introduced selection bias and limited statistical power. Given that subgroup analyses from RCTs, such as the study by Chapple et al. [50], reported comparable efficacy of treatments in reducing symptoms of refractory OAB between men and women, we deemed it methodologically acceptable to include studies with limited (<25%) male representation to minimize potential confounding.

In this study, we analyzed outcomes at the 3-month follow-up for the comparison of BoNT-A vs. placebo. A meta-analysis focusing on these 3-month outcomes was deemed appropriate for several reasons: first, the peak efficacy of intradetrusor BoNT-A injections is typically stable at 3 months, with effects lasting approximately 6–9 months [43,54,55]; second, placebo effects tend to wane by this time, enabling a clearer evaluation of the true treatment impact. For the BoNT-A vs. SNM comparison, all the included studies reported outcomes at the 6-month follow-up, which was thus selected for analysis, as neuromodulation effects are generally stabilized by this point [56]. The consistent reporting of 3- to 6-month outcomes across studies ensures adequate data for pooled analysis, making a meta-analysis over these timeframes a well-balanced approach that aligns with both clinical relevance and data availability.

### 5.3. Comparators

The meta-analysis focused on two key comparisons: intravesical BoNT-A vs. placebo and BoNT-A vs. SNM. The included studies utilized BoNT-A doses ranging from 100 IU to 300 IU. To ensure comparability of data across studies, a standardized extraction protocol was applied. For the BoNT-A vs. placebo comparison, outcome data were extracted exclusively from the 100 IU BoNT-A study arms. This dose was prioritized as it is the most extensively studied and recommended for initial treatment by major professional bodies, including the British Society of Urogynaecology (BSUG) [57], the American Urological Association/SUFU (AUA/SUFU) [58], and the UK National Institute for Health and Care Excellence (NICE) [59], with established efficacy–safety data. For the BoNT-A vs. SNM comparison, outcome data were extracted from the BoNT-A dose arms most frequently reported in the comparative studies (200 IU), as this was the dominant dose used in this specific study cohort.

Meta-analyses were conducted for those outcomes that were consistently reported across at least two primary studies in a comparable fashion.

Other treatment options for refractory OAB, such as percutaneous tibial nerve stimulation (PTNS), were excluded due to insufficient comparable data across studies, limiting the ability to yield relevant meta-analytic comparisons.

### 5.4. Data Extraction

A standardized data extraction form was utilized to collect relevant information from the studies included in the current meta-analysis. The data collected included study details: first author, publication year, study design, sample size, interventions, proportion of female participants, cost-effectiveness data and funding source. Data on prophylactic antibiotic use such as type of antibiotic, dose, route, and timing of administration were extracted where reported. Given the potential influence of antibiotics on post-procedural UTI rates, these data were collected to contextualize safety findings.

Two reviewers independently performed the data extraction, resolving any differences through discussion and consensus within the research team. BoNT-A was selected as the primary comparator due to its widespread use, while placebo and SNM were analyzed as distinct comparator groups. Studies evaluating the same interventions were grouped together to support effective pooled analyses.

### 5.5. Study Quality and Risk of Bias Assessment

Quality assessment of the included studies was conducted using the Critical Appraisal Skills Programme (CASP) tools [34,35], specifically the RCT [34] and cohort [35] study checklists. Both instruments provide a structured approach with guided questions to evaluate various aspects of primary studies. The CASP RCT tool [34] evaluates four key domains, focused on validity of results, methodological rigor, precision/comprehensiveness and local applicability. The CASP cohort tool [35] is structured across three sections, namely validity of results, their interpretation, and local utility.

Two researchers independently assessed all the included articles using the CASP tools (2024 versions), with discrepancies resolved through discussion within the research team.

Publication bias was not formally assessed, as fewer than 10 studies contributed to each outcome, below the recommended threshold of 10 for reliable analysis.

### 5.6. Data Analyses

All outcomes reported in the primary studies were extracted and centralized in a database. Meta-analyses were conducted for outcomes reported consistently across at least two primary studies. Outcomes were categorized as either dichotomous or continuous variables, with ORs used as the effect measure for dichotomous data and MDs for continuous data, both reported with 95% confidence intervals. Forest plots were generated using Review Manager 5.4.1 [60] to visually represent the results. Heterogeneity among studies was assessed using the I^2^ statistic, where a value below 50% indicated substantial homogeneity, prompting the use of fixed-effects models for pooled summary statistics. In cases of notable heterogeneity (I^2^ > 50%), random-effects models were applied to account for variability across studies. Sensitivity analyses were conducted for outcomes with contributions from at least three studies and substantial heterogeneity (I^2^ > 50%) using a leave-one-out approach, whereby individual studies were sequentially excluded to assess their influence on pooled estimates.

## Figures and Tables

**Table 1:** Characteristics of the included studies.

Study, Publication Year	Study Design	Interventions	Sample Size	Proportion of Female Participants (%)	Cost-Effectiveness Data Reported (Yes/No)	Funding Source
Al-Azzawi et al. [12], 2020	Prospective chort	BoNT-A (200 IU) vs. SNM	37	100%	Yes	None declared
Amundsen et al. [22], 2016	RCT	BoNT-A (200 IU) vs. SNM	381	100%	No	The Eunice Kennedy Shriver National Institute of Child Health and Human Development. The NIH Office of Research on Women’s Health at National Institutes of Health.
Amundsen et al. [23], 2018	RCT	BoNT-A (200 IU) vs. SNM	386	100%	No	The Eunice Kennedy Shriver National Institute of Child Health and Human Development. The NIH Office of Research on Women’s Health at National Institutes of Health.
Chermansky et al. [24], 2022	RCT	BoNT-A (100 IU) vs. Placebo *	115	90%	No	AbbVie
Denys et al. [25], 2012	RCT	BoNT-A (100 IU) vs. Placebo	54	87%	No	“Assistance Publique—Hôpitaux de Paris” Department of Clinical Research and Development), Grant of the French Ministry of Health (“Programme Hospitalier de Recherche Clinique”)
Dmochowski et al. [26], 2010	RCT	BoNT-A (100 IU) vs. Placebo	313	92%	No	AbbVie
Elmer-Lyon et al. [27], 2020	Retrospective cohort	BoNT-A (100-300 IU) vs. SNM	101	100%	No	None declared
Singh et al. [28], 2015	Retrospective cohort	BoNT-A (200 IU) vs. SNM	128	100%	No	None declared
Yokoyama et al. [29], 2020	RCT	BoNT-A (100 IU) vs. Placebo	248	75%	No	GlaxoSmithKline
Herschorn et al. [30], 2017	RCT	BoNT-A (100 IU) vs. Placebo	205	84%	No	Abb Vie
MacDiarmid et al. [31], 2023	RCT	BoNT-A (100 IU) vs. Placebo	120	93%	No	AbbVie
Nitti et al. [32], 2016	RCT	BoNT-A (100 IU) vs. Placebo	557	88%	No	None declared

* Both placebo and BoNT-A were combined with hydrogel and administered via bladder instillations.

**Table 2:** CASP criteria for RCTs.

CASP Criteria/Study	Amundsen et al., 2016 [22]	Amundsen et al., 2018 [23]	Chermansky et al., 2022 [24]	Denys et al., 2012 [25]	Dmochowski et al., 2010 [26]	Yokoyama et al., 2020 [29]	Herschorn et al., 2017 [30]	MacDiarmid et al., 2023 [31]	Nitti et al., 2016 [32]
1. Did the study address a clearly formulated research question?	y	y	y	y	y	y	y	y	y
2. Was the assignment of participants to interventions randomized?	y	y	y	y	y	y	y	y	y
3. Were all participants who entered the study accounted for at its conclusion?	y	y	y	n	y	y	y	y	y
4. (a) Were the participants ‘blind’ to intervention they were given?	n	n	y	y	y	y	y	y	y
4. (b) Were the investigators ‘blind’ to the intervention they were giving to participants?	n	n	y	y	y	y	y	y	y
4. (c) Were the people assessing/analyzing outcome/s ‘blinded’?	ct	ct	y	y	y	ct	ct	y	y
5. Were the study groups similar at the start of the randomized controlled trial?	y	y	y	y	y	y	y	y	y
6. Apart from the experimental intervention, did each study group receive the same level of care (that is, were they treated equally)?	y	y	y	y	y	y	y	y	y
7. Were the effects of intervention reported comprehensively?	y	y	y	y	y	y	y	y	y
8. Was the precision of the estimate of the intervention or treatment effect reported?	y	y	y	n	n	y	y	y	y
9. Do the benefits of the experimental intervention outweigh the harms and costs?	ct	ct	ct	ct	ct	ct	ct	ct	ct
10. Can the results be applied to your local population/in your context?	y	y	y	y	y	y	y	y	y
11. Would the experimental intervention provide greater value to the people in your care than any of the existing interventions?	ct	ct	n	y	y	y	y	y	y

Legend: y = yes, n = no, ct = cannot tell.

**Table 3:** CASP criteria for cohort studies.

CASP Criteria/Study	Al-Azzawi et al., 2020 [12]	Elmer-Lyon et al., 2020 [27]	Singh et al., 2015 [28]
1. Did the study address a clearly focused issue?	y	y	y
2. Was the cohort recruited in an acceptable way?	y	n	y
3. Was the exposure accurately measured to minimize bias?	y	y	y
4. Was the outcome accurately measured to minimize bias?	n	y	n
5. (a) Have the authors identified all important confounding factors?	n	n	y
5. (b) Have they taken account of the confounding factors in the design and/or analysis?	y	n	y
6. (a) Was the follow up of subjects complete enough?	y	y	y
6. (b) Was the follow up of subjects long enough?	n	y	n
7. What are the results of this study?	The procedures compared had similar safety and efficiency profiles.	UTI rates were similar in patients undergoing BoNT-A injections and SNM.	Treatment failure rates at six months were lower in the SNM group vs. BoNT-A group.
8. How precise are the results?	y	y	y
9. Do you believe the results?	y	y	y
10. Can the results be applied to the local population?	y	y	y
11. Do the results of this study fit with other available evidence?	n	y	y
12. What are the implications of this study for practice?	Patient preference, availability, cost, and surgical expertise should guide treatment choice, as both interventions have comparable efficacy but differ in invasiveness, cost/resource requirements, and durability of treatment.	Clinicians can reassure patients that UTI risk is comparable between BoNT-A and SNM. Recurrent UTI or prior prolapse repair increases UTI risk after either procedure.	SNM may be preferred over BoNT-A for short-term efficacy.

Legend: y = yes, n = no.

## Data Availability

No new data were created or analyzed in this study.

## Abbreviations

The following abbreviations are used in this manuscript:

OAB	Overactive bladder
UUI	Urgency urinary incontinence
UI	Urinary incontinence
UTI	Urinary tract infections
QoL	Quality of life
SNM	Sacral neuromodulation
BoNT-A	OnabotulinumtoxinA
PROs	Patient-reported outcomes
PROMs	Patient-reported outcome measures
RCT(s)	Randomized controlled trial(s)
PRISMA	Preferred Reporting Items for Systematic Reviews and Meta-Analyses
CENTRAL	Cochrane Central Register of Controlled Trials
BSUG	British Society of Urogynaecology
NICE	National Institute for Health and Care Excellence
AUA	American Urological Association
CASP	Critical Appraisal Skills Programme
TBS	Treatment Benefit Scale
KHQ	King’s Health Questionnaire
